# Comparison of the Ability to Control Water Loss in the Detached Leaves of *Wedelia trilobata*, *Wedelia chinensis*, and Their Hybrid

**DOI:** 10.3390/plants9091227

**Published:** 2020-09-18

**Authors:** Qilei Zhang, Guangxin Chen, Jundong Huang, Changlian Peng

**Affiliations:** Guangdong Provincial Key Laboratory of Biotechnology for Plant Development, School of Life Sciences, South China Normal University, Guangzhou 510631, China; 2014021299@m.scnu.edu.cn (Q.Z.); 2018010182@m.scnu.edu.cn (G.C.); 2019022493@m.scnu.edu.cn (J.H.)

**Keywords:** antioxidant activity, abscisic acid, biological invasion, drought stress, hybridization

## Abstract

In the process of biological invasion, hybridization between invasive species and native species is very common, which may lead to the formation of hybrids with a stronger adaptability. The hybrid of *Wedelia trilobata* (an alien invasive species) and *Wedelia chinensis* (an indigenous congener) has been found in South China. In our previous study, we found that the hybrid showed heterosis under cadmium stress. However, the results of this experiment demonstrated that the leaves of the hybrid had no heterosis in controlling water loss. The results showed that the water loss rate of *W. trilobata* was the slowest, that of *W. chinensis* was the fastest, and that of the hybrid was in the middle. Compared with *W. chinensis* and the hybrid, *W. trilobata* accumulated more abscisic acid (ABA) in leaves to control water loss. After the leaves were detached, *W. chinensis* leaves suffered the most serious damage, the lowest maximum photochemical efficiency, the most serious membrane lipid peroxidation, and the largest accumulation of malondialdehyde and reactive oxygen species. Compared with *W. chinensis* and its hybrid, the leaves of *W. trilobata* could accumulate more antioxidant enzymes and antioxidants, and the total antioxidant capacity was the strongest. The results demonstrate that the ability of the hybrid to reduce water loss was lower than that of *W. trilobata*, but higher than that of *W. chinensis*. They showed that the drought resistance of the hybrid may be higher than that of *W. chinensis*, and it might threaten the survival of *W. chinensis*.

## 1. Introduction

In the process of plant growth, organisms are subjected to various adverse environmental stresses, among which drought is one of the most common abiotic stresses. The effects of drought stress on plants are mainly reflected in the effects on cell activity and organ and tissue function [1,2]. A decrease in the water content will result in the inhibition of photosynthesis, the slow growth of plants, and influence of the biomass or yield [3]. After drought stress, the metabolic process of cells is blocked, which leads to the accumulation of H_2_O_2_, ^1^O_2_, and O_2_^• −^ in cells [4]. The main component of the cell membrane is the phospholipid bilayer. A large number of reactive oxygen species will damage the properties of the cell membrane. The results of membrane lipid peroxidation are mainly an increase in the malondialdehyde (MDA) content and an increase in the cell membrane permeability [5]. Due to the destruction of the cell membrane and the increase in the permeability, the cell membrane will lose the function of selective passage, resulting in uncontrolled material passing in and out of the cell, resulting in the disorder of the cell metabolic function.

In the face of drought stress, plants have formed a series of strategies, the first of which is to reduce water loss. Most water loss (transpiration) is produced through stomata. Therefore, reducing stomatal opening or closing is key to reducing water loss under drought conditions [6]. Abscisic acid (ABA), as an endogenous plant hormone, is closely related to stomatal opening and closing, especially under drought stress [7]. ABA can be used as a signal substance to change the ion concentration inside and outside of the guard cells, thus causing stomatal movement [8]. Some studies have shown that the ABA content in plant cells increased significantly under drought stress. Exogenous ABA or the overexpression of ABA can increase the ability of plants to resist drought stress [9,10]. The second is the strategy to increase the osmotic substances in the cells. The ability of osmoregulation is considered to be among the criteria for the drought resistance of plants. There are many osmoregulants in plant cells, among which proline is the most studied so far. A large amount of data has shown that there is a positive correlation between proline accumulation and plant drought stress [11]. Proline can stabilize the membrane and protein structure, scavenge reactive oxygen species, and regulate cytoplasmic pH under drought stress [12,13]. The other strategy is to increase the antioxidant capacity of cells. Oxidative stress usually occurs simultaneously with drought stress. Aerobic metabolism provides energy for plant growth and development, but it is also accompanied by the production of reactive oxygen species (ROS) [4]. Under ROS stress, the spatial structures of various membrane proteins or enzymes are disturbed, resulting in an increased membrane permeability and ion leakage, chlorophyll damage, metabolic disorders, and even serious injury or death of plants. In order to protect cells from the harmful effects of excessive ROS, plants have evolved a series of complex enzymatic and nonenzymatic antioxidant defense mechanisms to maintain the homeostasis of the intracellular redox state. Protective enzymes include superoxide dismutase (SOD), catalase (CAT), and peroxidase (POD). Nonenzymatic antioxidants include vitamins, phenols, and ketones. Under drought stress, the content of antioxidants in plants increases significantly [14].

Biological invasion has become increasingly common. In the process of biological invasion, the phenomenon of hybridization between invasive species and native species often occurs. Interspecific hybridization is a common phenomenon in plants. It has been reported that at least 25% of plant species have undergone hybridization or gene penetration with other species [15]. Hybridization between native and invasive species usually results in new species, which leads to genetic variation, a fixation of heterosis, and a reduction in the genetic load, thus promoting the evolution of invasiveness and adaptability [16]. Therefore, hybrids formed by hybridization between invasive species and native species usually have a higher adaptability and wider ecological range than their parents [16,17]. The hybridization between native species and invasive species will reduce the genetic diversity and population diversity of native species, especially for endangered species. Sometimes, the diffusion of hybrids will reduce the biomass of native species and even cause the extinction of native species [18,19].

*Wedelia trilobata* (L.) Pruski and *Wedelia chinensis* (Osbeck) Merr. belong to the *Asteraceae* family. They are creeping perennial herbal plants found in South America and South China. A hybrid of *W. trilobata* and *W. chinensis* was identified in the wild in 2013 [20]. A previous study showed that the response of the hybrid to nitrogen deposition and the tolerance of the hybrid to weak light and low temperature stress have no heterosis compared with its parents [21,22]. Our recent research demonstrated that hybridization between *W. trilobata* and *W. chinensis* significantly improved the tolerance of the hybrid to cadmium stress [23]. However, the stress tolerance of this hybrid to drought stress exposure remains largely unknown. The leaf is the most important organ of water loss in plants. This study aimed to investigate the water loss rate of detached leaves of *W. chinensis*, *W. trilobata*, and their hybrid.

## 2. Results

### 2.1. Changes of Water Loss in Detached Leaves

**Figure 1:**
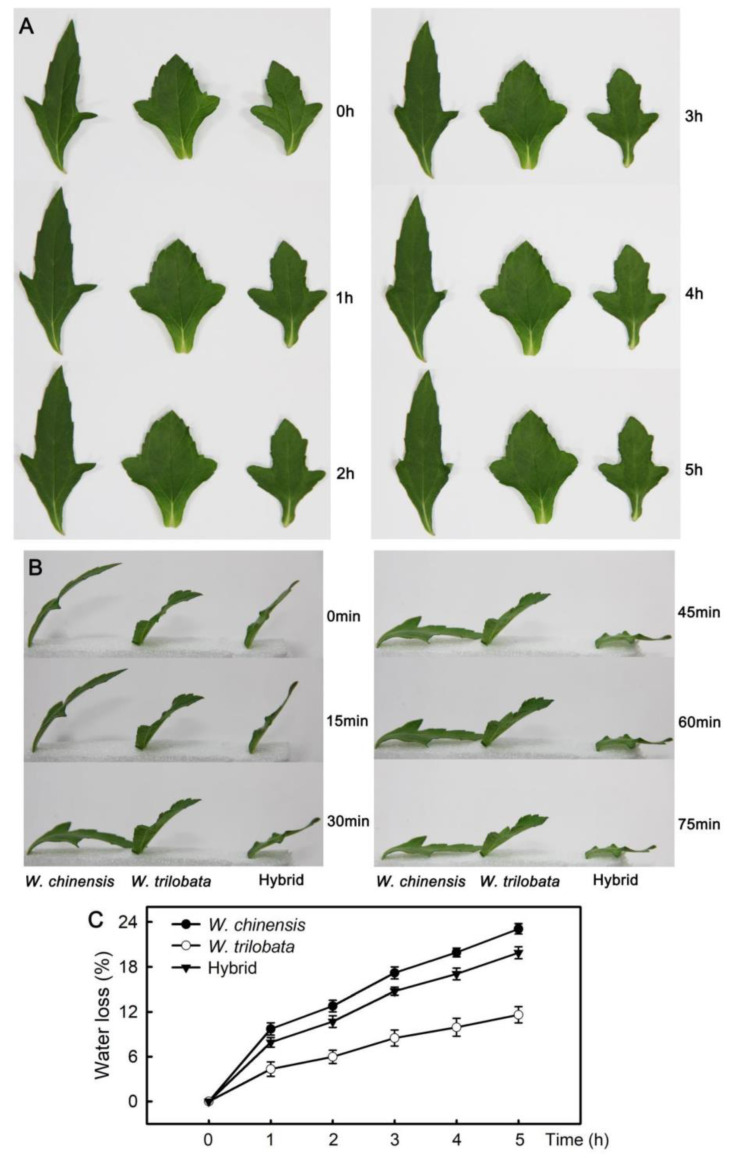
Phenotype and water loss. Phenotypic changes of detached leaves (**A**,**B**). Comparison of the water loss rate of *Wedelia chinensis*, *Wedelia trilobata*, and their hybrid of detached leaves (**C**). The data are shown as means ± standard errors (SE), and the study included five biological replicates.

The leaf phenotype showed that the leaves of *W. chinensis*, *W. trilobata*, and their hybrid exhibited wilting with the prolongation of the leaf in vitro time, among which the *W. chinensis* displayed the most obvious performance (Figure 1A,B), and the leaf edge angle had curled at 3 h (Figure 1A). Figure 1C shows the changes of leaf water loss for the three plants. With the extension of the in vitro time, the water loss rate of leaves of *W. chinensis* was the highest, and that of *W. trilobata* was the lowest. The water loss rate of hybrid leaves was between those of its parents.

### 2.2. Abscisic Acid Content and Related Gene Expression in Detached Leaves

**Figure 2:**
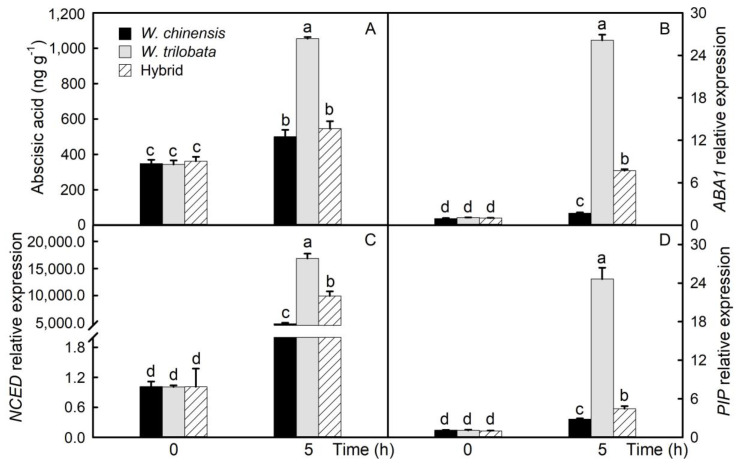
Abscisic acid and gene expression. Abscisic acid content in detached leaves at 0 and 5 h (**A**). Relative expression of *ABA1*, *NCED*, and *PIP* genes in detached leaves at 0 and 5 h (**B**–**D**). The data are shown as means ± standard errors (SE), and the study included five biological replicates. Different letters (a, b, c, d) on the bar represent significant differences at *p* < 0.05 (Duncan’s test).

The ABA content of detached leaves increased significantly. The ABA content of *W. trilobata* was significantly higher than that of *W. chinensis* and its hybrid. The content of ABA increased by 43%, 207%, and 51% in *W. chinensis*, *W. trilobata*, and their hybrid, respectively (Figure 2A). *ABA1* and *NCED* are the main regulatory genes in the ABA synthesis pathway. The relative expression levels of *ABA1* and *NCED* in detached leaves were significantly up-regulated, and their trend was consistent (Figure 2B,C). The results showed that the relative expression of *ABA1* and *NCED* in the leaves of *W. trilobata* was significantly higher than that of hybrids, and that of hybrids was significantly higher than that of *W. chinensis*. *PIP* is a key gene in the synthesis of aquaporin, and the relative expression of the *PIP* gene in detached leaves also increased significantly (Figure 2D). Moreover, the trend was consistent with that of *ABA1* and *NCED*.

### 2.3. Changes of Chl Fluorescence and the Localization of Reactive Oxygen Species in Detached Leaves

**Figure 3:**
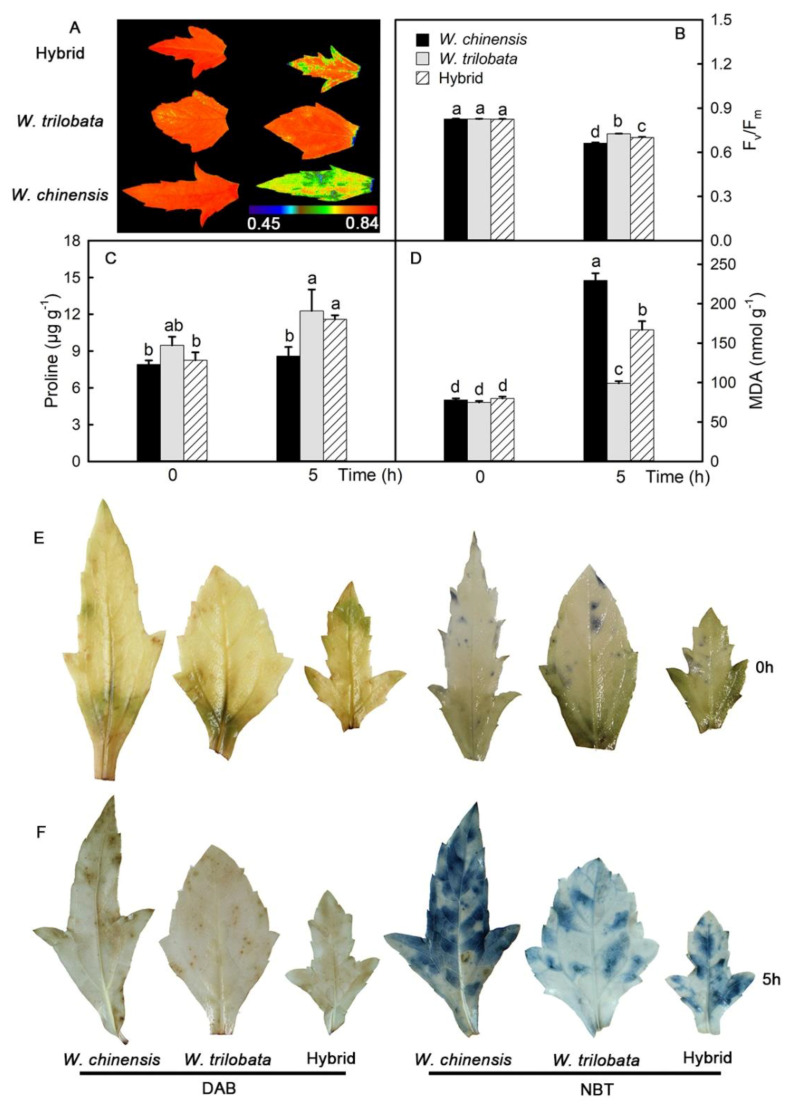
Maximum photochemical efficiency (Fv/Fm) and reactive oxygen species. Fv/Fm of detached leaves at 0 and 5 h (**A**,**B**). Proline content in detached leaves (**C**). Malondialdehyde (MDA) content of detached leaves at 0 and 5 h (**D**). Accumulation of hydrogen peroxide (DAB) and superoxide cation (NBT) in detached leaves at 0 and 5 h (**E**,**F**). The data are shown as means ± standard errors (SE), and the study included five biological replicates. Different letters (a, b, c, d) on the bar represent significant differences at *p* < 0.05 (Duncan’s test).

In detached leaves, the maximal photochemical efficiency (Fv/Fm) of *W. chinensis* leaves was the lowest, and that of *W. trilobata* was the highest (Figure 3A,B). Proline, as an osmoregulation substance, can regulate the osmotic pressure of cells, and the proline content of detached leaves increased (Figure 3C). MDA is a product of membrane lipid peroxidation, which can reflect the degree of cell membrane damage. The content of MDA in leaves increased significantly, among which *W. chinensis* had the highest content, increasing by 194%, while *W. trilobata* had the lowest content, with an increase of 32%. The content of the hybrid was between that of its parents, increasing by 108% (Figure 3D). It was found that the accumulation of ROS in detached leaves increased, and the accumulation of ROS in leaves of *W. trilobata* was the lowest (Figure 3E,F).

### 2.4. Changes of the Antioxidant Capacity in Detached Leaves

**Figure 4:**
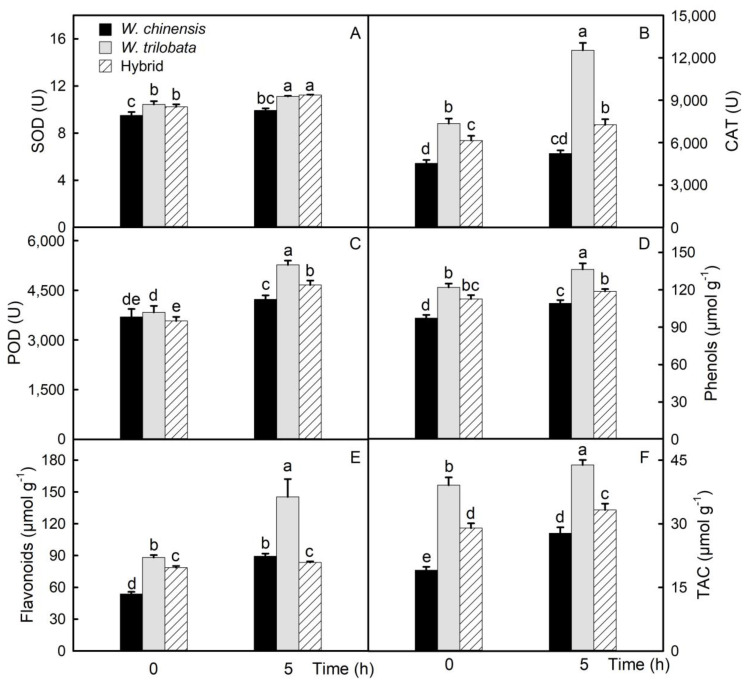
Antioxidants. The activity of superoxide dismutase (SOD), catalase (CAT), and peroxidase (POD) of detached leaves at 0 and 5 h (**A**–**C**). The contents of total phenols and flavonoids of detached leaves at 0 and 5 h (**D**,**E**). The total antioxidant capacity (TAC) of detached leaves at 0 and 5 h (**F**). The data are shown as means ± standard errors (SE), and the study included five biological replicates. Different letters (a, b, c, d, e) on the bar represent significant differences at *p* < 0.05 (Duncan’s test).

Plants can reduce oxidative stress in cells by synthesizing antioxidants. The contents of SOD, CAT, and POD were increased significantly in detached leaves. The contents of SOD, CAT, and POD in *W. trilobata* were the highest, and those of *W. chinensis* were the lowest (Figure 4A–C). Total phenols and flavonoids were nonenzymatic antioxidants, and their contents also increased significantly in detached leaves (Figure 4D,E). Furthermore, the trend was consistent with SOD, CAT, and POD. The total antioxidant capacity (TAC) reflected the antioxidant capacity of nonenzymatic antioxidants. The content of total antioxidant substances in the leaves of *W. trilobata* was significantly higher than that of the hybrid, and that of the hybrid was higher than that of *W. chinensis* (Figure 4F).

## 3. Discussion

The hybrid leaves showed no heterosis in controlling water loss. As the main organ of water loss in plants, most of the water is lost through leaves. Therefore, the ability of plant leaves to control water loss can reflect the drought resistance of plants. In this paper, the water loss rate of detached leaves of *W. chinensis*, *W. trilobata*, and their hybrid was compared. The results showed that the water loss rate of leaves of *W. trilobata* was the lowest, and that of leaves of *W. chinensis* was the highest. The water loss rate of hybrid leaves was between that of their parents, and it was closer to that of *W. chinensis*. The phenotype of detached leaves showed that *W. chinensis* leaves wilted first. Water loss in leaves mainly occurs through the stomata on the leaves, and the stomatal opening is positively correlated with the water loss of plants. Studies have shown that under drought stress, plants will reduce stomatal opening or close stomata to reduce water loss and improve drought resistance [24,25]. Many studies have shown that ABA plays a key role in stomatal regulation. The exogenous application of ABA or endogenous increase in ABA expression can cause stomata to close, thus reducing water loss [26,27]. The results showed that the ABA content of detached leaves increased significantly, and that of *W. trilobata* was significantly higher than that of *W. chinensis* and hybrids, which was consistent with the results of water loss. In *Arabidopsis thaliana*, it was found that the overexpression of ABA synthesis-related genes could increase the ABA content in leaves, reduce the water loss of detached leaves, and improve the drought resistance of plants [26]. *ABA1* and *NCED* are the main regulatory genes in the ABA synthesis pathway, and their expression levels are positively correlated with the ABA content. In *Arabidopsis*, drought can increase the expression of *NCED* and the content of ABA. Compared with wild type specimens, the ABA content in ABA-deficient mutants decreased, and drought resistance decreased under drought stress [28,29]. The results of this study showed that the expression of *ABA1* and *NCED* in detached leaves increased significantly. The expression level of *ABA1* and *NCED* in *W. trilobata* was the highest, and that in *W. chinensis* was the lowest. *PIP* is a key gene in the synthesis of aquaporin. The increase in *PIP* expression can increase the content of aquaporin. Aquaporin is the most important aquaporin on the cell membrane, and can affect the water transport and regulation between cells. Figure 2 shows that the *PIP* expression increased significantly, which was consistent with *ABA1* and *NCED* expression. Studies have shown that increasing the content of aquaporin in leaves can improve the drought resistance of plants [30].

Fv/Fm is one of the most important parameters of chlorophyll fluorescence in plant leaves, and its value will not change significantly under natural conditions. However, it will decrease under stress (high temperature, low temperature, drought, etc.) [31,32]. The results of this study showed that the Fv/Fm of leaves decreased significantly, indicating that the detached leaves were damaged. Among them, *W. chinensis* leaves exhibited the lowest values, while those of *W. trilobata* were the highest. It was suggested that the leaves of *W. trilobata* were the least damaged, while the leaves of *W. chinensis* were the most seriously damaged. Drought stress is often accompanied by oxidative stress, and the accumulation of ROS in plants increases significantly. It was found that drought could increase the accumulation of ROS in rice leaves [25]. The results demonstrated that the accumulation of ROS in detached leaves increased. A large amount of ROS can cause toxicity to plant cells, which can destroy biological macromolecules, attack cell membranes, and accelerate leaf damage [33]. In this experiment, the accumulation of ROS in the leaves of *W. chinensis* was the highest, and that in the leaves of *W. trilobata* was the lowest, indicating that the leaves of *W. chinensis* were under the most serious oxidative stress. MDA is a product of membrane lipid peroxidation and can indicate the damage of stress to plants. The content of MDA and the change of plasma membrane permeability are important indicators reflecting the degree of membrane lipid peroxidation and plasma membrane damage. In the study of *Zoysia japonica*, it was found that with the extension of the drought time, the content of MDA in leaves increased gradually [34]. The results revealed that the content of MDA in detached leaves increased significantly, indicating that the detached leaves were damaged. Among them, the content of MDA in leaves of *W. chinensis* was the highest, and that of *W. trilobata* was the lowest, indicating that the leaves of *W. chinensis* were the most seriously damaged.

In the long-term evolution of plants, the mechanism of stress adaptation is restricted by heredity. On the one hand, plants scavenge reactive oxygen radicals by enhancing the activities of antioxidant enzymes (SOD, CAT, and POD), in order to maintain the stability and integrity of the cell membrane [35]. The results showed that the activities of SOD, CAT, and POD in detached leaves of *W. chinensis*, *W. trilobata*, and their hybrid increased significantly. Among them, the enzyme activity in the leaves of *W. trilobata* was the strongest, the enzyme activity in the leaves of *W. chinensis* was the weakest, and the enzyme activity in the leaves of the hybrid was between that of its parents. A previous study showed that increasing the activities of SOD, CAT, and POD could significantly reduce the accumulation of ROS in plant leaves [23]. On the other hand, plants can also accumulate nonenzymatic antioxidants to improve the antioxidant capacity and scavenge ROS. These nonenzymatic antioxidants include vitamins, phenols, and flavonoids [36]. Studies have shown that increasing the contents of total phenols and flavonoids in plant leaves can reduce the accumulation of reactive oxygen species under stress [23]. The results of this study showed that the contents of total phenols and flavonoids in detached leaves of *W. chinensis*, *W. trilobata*, and their hybrid increased significantly, and the trend was consistent with enzyme activity.

## 4. Materials and Methods

### 4.1. Plant Materials

*W. trilobata* is one of the invasive species in South China, *W. chinensis* is a native species in South China, and their hybrid has been found in the field. *W. trilobata*, *W. chinensis*, and their hybrid were propagated asexually and planted in the experimental field of the biological garden of South China Normal University, Guangzhou, China. The annual average temperature and rainfall are 22.8 °C and 1736 mm, respectively, belonging to the subtropical humid monsoon climate [37]. Mature leaves at the fourth leaf position were selected as research materials. Five replicates were performed.

### 4.2. Water Loss Assay

The detached leaves were incubated at room temperature and weighed at 60 min intervals. The water loss (%) was calculated by dividing the difference between the initial fresh weight and the postincubation fresh weight by the initial fresh weight.

### 4.3. Hormone Determination

In this study, 0.1 g leaves were weighed and ground with 0.5 mL PBS (50 mM; pH 7.3) on ice and then left at 4 °C for 2 h. The grinding solution was centrifuged at 4 °C for 20 min at 5000× *g*, and the supernatant was the hormone extract. The contents of the hormone were determined by a plant ELISA Kit (Zike, Shenzhen, China).

### 4.4. Gene Expression Analysis

The analysis of gene expression was conducted according to the method published by Zhang et al. [38]. Normalization and fold changes were calculated using the methods of Livak and Schmittgen [39]. The *GAPDH* gene was used as an internal reference; the primer pairs were 5′-CTGCTTCATTCAACATC-3′ (forward) and 5′-CTCACGGTCAGATCAACA-3′ (reverse). The primer pairs for the *ABA1* gene were 5′-AACCGAGTCTCCAAGCAAGG-3′ (forward) and 5′-CCAGGTAGAAAGGAACGGCT-3′ (reverse). The primer pairs for the *NCED* gene were 5′-ACGTCGTTTTCCTGCGAGAT-3′ (forward) and 5′-GCATGGGAAGAACCCGATCA-3′ (reverse). The primer pairs for the *PIP* gene were 5′-GCTCCCAAGCCTCAGGAAAT-3′ (forward) and 5′-TTCCGGAATGGCAGGTGTTT-3′ (reverse).

### 4.5. Determination of Enzyme Activity

In this investigation, 0.2 g leaves were homogenized in 1.5 mL phosphate buffer (50 mM) containing 2% (w/v) polyvinylpyrrolidone, 0.1% (v/v) Triton-X-100, and 100 mM ethylenediaminetetraacetic acid disodium salt (EDTA-Na_2_). The grinding fluid was centrifuged at 13,000× *g* for 15 min at 4 °C, and the resulting supernatant was retrieved as an enzyme extract.

The activity of POD was evaluated according to the method described by Chance and Maehly [40], with moderate adjustments. In total, 0.1 mL of the enzyme extract was mixed with 1.875 mL phosphate buffer (50 mM; pH 7.0), 1 mL H_2_O_2_ (30 mM), and 0.025 mL 2-methoxyphenol. The absorbance was recorded nine times (20 s each time) at 470 nm (*UV-2450*, Shimadzu, Kyoto, Japan). The activity of POD (1 unit) was estimated as the quantity of enzyme needed for a 0.01-unit increase in OD within 1 min.

The activity of SOD was evaluated according to the method published by Giannopolitis and Ries [41], with moderate adjustments. In total, 0.1 mL of the enzyme extract was mixed with 1.7 mL phosphate buffer (50 mM; pH 7.8), 0.3 mL methionine (130 mM), 0.3 mL NBT (0.75 mM), 0.3 mL EDTA-Na_2_ (0.1 mM), and 0.3 mL riboflavin (0.02 mM). The reaction was conducted at a photosynthetic photon flux density of 4500 Lux for 15 min. Subsequently, the absorbance was determined immediately at 560 nm (*UV-2450*, Shimadzu, Kyoto, Japan) after the reaction was terminated in the dark. The amount of enzyme that resulted in 50% suppression of the photochemical reduction in NBT was defined as the unit of enzyme activity.

The activity of CAT was evaluated according to the method described by Chance and Maehly [40], with moderate adjustments. A total of 0.1 mL of the enzyme extract was mixed with 2.9 mL H_2_O_2_ (30 mM). After 15 s of incubation, the absorbance was recorded nine times (20 s each time) at 240 nm (*UV-2450*, Shimadzu, Kyoto, Japan). The activity of CAT (1 unit) was estimated as the quantity of enzyme needed for a 0.01-unit increase in the optical density (OD) within 1 min.

### 4.6. Determination of Total Phenols and Flavonoids

The total phenols were determined by the Folin–Ciocalteu method, as described by Ainsworth and Gillespie [42]. In the procedure, 0.05 g leaves were cut into pieces and placed in a centrifuge tube containing 1.5 mL 95% methanol and extracted at 4 °C for 24 h. Then, 1 mL 10% Folin–Ciocalteu and 2 mL 0.7 M Na_2_CO_3_ were mixed and added to a 0.5 mL sample. The absorbance was measured at 765 nm (*UV-2450*, Shimadzu, Kyoto, Japan), and the concentration was calculated according to the standard curve of gallic acid.

The flavonoids were determined according to the method described by Heimler et al. [43]. In this procedure, 0.05 g leaves were cut into pieces and placed in a centrifuge tube containing 1.5 mL 95% methanol and extracted at 4 °C for 24 h. The 0.15 mL sample was mixed with 1.85 mL deionized water, 0.2 mL 5% NaNO_2_, 0.3 mL 10% AlCl_3_ (freshly prepared), and 1 mL 1 M NaOH. The absorbance was measured at 510 nm (*UV-2450*, Shimadzu, Kyoto, Japan), and the concentration was calculated according to the standard curve of catechin.

### 4.7. Determination of Total Antioxidant Capacity

The total antioxidant capacity (TAC) of leaves was evaluated by measuring the rate of 1,1-diphenyl-2-picrylhydrazyl (DPPH) scavenging [44]. In this procedure, 0.05 g leaves were cut into pieces and placed in a centrifuge tube containing 1.5 mL 95% methanol and extracted at 4 °C for 24 h. The 0.3 mL sample was mixed with 2.7 mL 120 μM DPPH. The absorbance was measured at 517 nm (*UV-2450*, Shimadzu, Kyoto, Japan), and the concentration was calculated according to the standard curve of DPPH.

### 4.8. Detection of Chl Fluorescence

Chl fluorescence was measured by the Chl fluorescence imaging system (Technologica, Essex, UK). The minimum fluorescence (F0) and the maximum fluorescence (Fm) of the leaves and stems were measured using a 6000 μmol m^−2^ s^−1^ saturating pulse. The maximum photochemical efficiency (Fv/Fm) was calculated as Fv/Fm = (Fm − F0)/Fm [45].

### 4.9. Determination of Proline

A 0.2 g sample was ground with 10 mL 80% ethanol, and the homogenate was then extracted with 0.01 g activated carbon in the dark for 1 h. The extract was filtered, 1 g zeolite was added, and the sample was shaken for 15 min and centrifuged for 5 min at 3000× *g*. Then, 2 mL supernatant, 2 mL acetic acid, and 2 mL ninhydrin solution (1.25 g of ninhydrin was dissolved in 30 mL acetic acid and 20 mL 6 M of phosphoric acid) were mixed in boiling water for 20 min. The absorbance of the solution was measured after cooling at 517 nm (*UV-2450*, Shimadzu, Kyoto, Japan), and the concentration was calculated according to the standard curve of proline.

### 4.10. Determination of Malondialdehyde

The MDA was measured according to the method published by Sun et al. [22]. A 0.2 g sample was homogenized with 2 mL 10% trichloroacetic acid. After centrifuging at 4000× *g* for 15 min at 4 °C, the supernatant (1 mL) was mixed with an equal volume of 0.67% 2-thiobarbituric acid and then boiled for 20 min. The absorbance of the solution was measured after cooling at 600, 532, and 450 nm using a *UV-2450* spectrophotometer (Shimadzu, Tokyo, Japan).

### 4.11. Tissue Localization of Reactive Oxygen Species

The tissue localization of H_2_O_2_ was conducted according to the method described by Liu et al. [46]. Leaves were placed in 0.2 M phosphate buffer (pH 7.0) containing 1 mg mL^−1^ 3,3′-diaminobenzidine (DAB) for 2 h at room temperature after brief vacuum infiltration three times (10 min each), and the leaves were then decolored in 95% ethanol.

The tissue localization of O_2_^•−^ was conducted according to the method described by Liu et al. [46]. Leaves were placed in 0.2 M phosphate buffer (pH 6.4) containing 1 mg mL^−1^ nitroblue tetrazolium (NBT) and 10 mM NaN_3_ for 4 h at room temperature after brief vacuum infiltration three times (10 min each), and the leaves were then decolored in 95% ethanol.

### 4.12. Statistical Analysis

SPSS Statistics 19.0 (IBM, New York, NY, USA) software was used to analyze the data. Data were subjected to one-way analysis of variance (ANOVA) with Duncan’s multiple range test to compare means, and significance set at two-tailed *p* < 0.05. All analyses were conducted using SigmaPlot version 12.5 (SYSTAT Software, Richmond, CA, USA).

## 5. Conclusions

In summary, the ability of the hybrid of *W. trilobata* and *W. chinensis* to control water loss in leaves was weaker than that of the invasive species *W. trilobata*, but stronger than that of the native species *W. chinensis*. In the process of leaf water loss, the water loss rate of *W. trilobata* was the lowest, and that of *W. chinensis* was the highest. The antioxidant capacity of *W. trilobata* was the strongest, while that of *W. chinensis* was the weakest. *W. chinensis* suffered the most serious oxidative stress, while *W. trilobata* suffered the least. The results indicate that the drought resistance of the hybrid is not superior to that of its parents but is stronger than that of the native species. The survival of native species is threatened not only by invasive species but also by hybrids. Therefore, the emergence of hybrids may accelerate the extinction of native species.
